# Differences in nutritional intake from diet and nutritional supplements between urban and rural pregnant women in China: a nationwide cross-sectional study

**DOI:** 10.3389/fnut.2025.1634739

**Published:** 2026-01-14

**Authors:** Minghan Fu, Yun Zhou, Jia Yin, Zhencheng Xie, Qin Zhang, Hongliang Luo, Yitong Li, Jiahui Huang, Zhixu Wang, Ye Ding

**Affiliations:** 1 Department of Maternal, Child and Adolescent Health, School of Public Health, Nanjing Medical University, Nanjing, China; 2 Danone Open Science Research Center for Life-Transforming Nutrition, Shanghai, China; 3 The Institute of Nutrition and Food Science, Nanjing Medical University, Nanjing, China

**Keywords:** dietary assessment, food atlas, nutritional supplements, pregnancy, urban–rural disparities

## Abstract

**Introduction:**

Maternal nutritional status during pregnancy affects pregnancy outcomes and the lifelong health of mothers and offspring. In China, prenatal dietary habits and nutritional supplement usage vary across geographic regions, socioeconomic strata, and dietary cultures. This study aimed to evaluate and compare dietary and nutrient intakes of urban and rural pregnant women in China, providing baseline data for relevant health guidance and strategies.

**Methods:**

This multi-stage cross-sectional survey recruited 653 healthy urban and rural pregnant women in their second or third trimesters from 2 municipalities and 11 provinces in China. Dietary and nutritional supplement consumption was assessed using a 4-day online diary paired with a food atlas. The total daily intake of each food category, energy, and key nutrients was evaluated based on the Chinese balanced dietary pagoda and the Chinese Dietary Reference Intakes. The Mann–Whitney U test and Chi-square test were utilized to compare urban–rural differences.

**Results:**

In this study, both urban and rural pregnant women in different trimesters exhibited an inadequate consumption of multiple food categories: potatoes, vegetables, fruits, fish, shrimp, and shellfish, eggs, milk and its products, soybeans and its products, and nuts, while the intake of livestock meat and poultry, and cooking oil were excessive. Energy and protein intake were inadequate, with imbalanced energy contributions of carbohydrates and fats. Micronutrient deficiencies were widespread, with 13.5 ~ 99.2% of individuals falling below their estimated average references, particularly for VA, VB_1_, VB_6_, VB_9_, calcium, magnesium, and iodine. These nutritional challenges were more severe in rural areas than in urban areas.

**Discussion:**

In conclusion, dietary intake of both urban and rural pregnant women in China is highly imbalanced. Even with nutritional supplements in the survey, many pregnant women lack essential nutrients. A scientific nutrition plan should be developed for them, and a diverse and balanced diet is crucial, especially in rural areas.

## 1 Introduction

The nutritional status during pregnancy not only affects pregnancy outcomes, but is also closely related to maternal health, fetal physical and intellectual development, and the health of offspring in adulthood (1). By improving dietary structures and implementing scientific nutritional interventions such as nutrient fortified foods and supplements, it is possible to effectively mitigate health issues associated with inadequate, excessive, or imbalanced intake of food, energy and nutrients, thereby providing more comprehensive health protection for both mothers and children (2, 3).

The dietary survey is a pivotal tool in comprehensively assessing the dietary structures, as well as the quantity and quality of energy and nutrients intake among pregnant women over a specific period. Accurate collection of dietary intake data is essential for informing health and nutrition policies. Retrospective dietary survey methods, such as 24-h recall and food frequency method, are the most widely used methods for collecting dietary information, depending on respondents’ ability to recall and describe their food consumption before the survey (4). The accuracy of dietary data tends to decline as the time interval extends between the meal intake and the subsequent dietary recall, with errors becoming increasingly pronounced over time (5). Additionally, these retrospective methods are often based on respondents’ self-assessment of their food consumption, demanding a certain level of educational proficiency and practical life experience to ensure the accuracy and reliability of the data provided (6). However, in the general population’s mindset, there is often a disconnect between the visual perception of various foods and their actual weight, making precise estimation of food weight a significant hurdle in dietary data collection.

So far, the application of specialized dietary survey methods to study the dietary nutritional status of Chinese pregnant women have mainly focused on specific groups such as rural areas and ethnic minorities, or have been limited to certain local areas such as western areas and eastern coastal cities, or have only focused on single nutrients such as iodine or iron (7–9). The dietary status during pregnancy exhibited considerable variation across different Chinese geographic locations, socioeconomic strata, and diverse dietary cultures. For instance, in Urumqi, Xinjiang Uygur Autonomous Region, the consumption of staple foods, vegetables, milk and its products, as well as fish, shrimp, and shellfish among pregnant women were inadequate (10). Conversely, in Suzhou, Jiangsu Province, there were over-consumption of staple foods and fruits, while the consumption of milk and its products was not adequate (11). Therefore, it is necessary to carry out dietary surveys in multiple geographic regions and among different socioeconomic groups simultaneously to obtain more reliable and nationally representative data.

Currently, the only nationwide dietary survey is the China Health and Nutrition Survey (CHNS) (12), but unfortunately, it does not take into account the consumption of nutritional supplements. In China, various domestic or imported nutritional supplements have been introduced to the market, which are commonly used to improve the nutritional intake of individuals with nutrient deficiencies. A previous survey conducted in three Asian-Pacific countries revealed a concerning phenomenon: in China, 46.0, 42.0, and 39.0% of pregnant women (with a sample size of 600) took multiple vitamins during the first, second, and third trimesters of pregnancy, respectively (13). This highlights the necessity of considering the potential impact of nutritional supplements on the nutritional status of pregnant women.

To overcome these limitations, using an online dietary diary method featuring a food atlas embedded with three visual reference systems (6), this study collected baseline data on diet and nutritional supplements through a multi-regional cross-sectional survey to assess the dietary structures, energy, and nutrient intake of Chinese pregnant women during the second and third trimesters, to identify risks of deficiencies, excesses, or imbalances in energy and nutrients. In addition, with the acceleration of China’s urbanization process and significant improvement in living standards, rural residents are struggling to cope with ongoing challenges, including low income levels, limited education levels, traditional ways of thinking, and inadequate transportation infrastructure, all of which will have an impact on their food supply capacity and dietary behavior. Therefore, this study further compared the differences between urban and rural areas, which will help provide appropriate dietary guidance and recommendations for pregnant women in different regions.

## 2 Methods

### 2.1 Study participants

Our study was founded upon a dietary survey that targeted pregnant women in their second and third trimesters in China (2018–2019), which was approved by the Ethics Committee of Nanjing Medical University (Nanjing, China) with approval number 2018–1123. This survey was a cross-sectional design using a multi-stage sampling method. To begin with, 2 municipalities (Beijing and Shanghai) and 11 provinces (Liaoning, Hebei, Henan, Anhui, Hubei, Jiangsu, Sichuan, Yunnan, Zhejiang, Guangdong and Fujian) were selected based on geographical location, economic status, regional population and birth rate. Subsequently, urban and rural areas were delineated from each municipality or province, and a representative city from each area was selected for the survey. Finally, participants were randomly recruited from each city, based on the information provided by the maternal and child health centers in the selected cities. Before the face-to-face interviews, participants were initially contacted via phone to establish the location for data collection. The participants were presented with detailed information of the survey and signed an official informed consent form.

The inclusion criteria specified healthy pregnant women between the ages of 20 and 44 who had a singleton pregnancy in the second trimester (13 ~ 27 weeks) or the third trimester (28 weeks to pre-delivery). They were not clinically diagnosed with infectious diseases, metabolic disorders (such as obesity, diabetes and hypertension), pregnancy complications (such as hyperemesis gravidarum, gestational diabetes and hypertensive disorder complicating pregnancy) and nutritional disorders (such as anemia, iodine deficiency goiter and osteoporosis). Pregnant women with limited cognitive capacity who were unable to report their dietary intake, as well as those who underwent *in vitro* fertilization, chemotherapy, or were involved in other studies that required food restriction, were excluded from the study.

The formula $N=\frac{{\left(Z_{\alpha }\times S\right)}^{2}}{d^{2}}$ was used to determine the sample size (14). Z represents the desired confidence interval, which was established at 95% (*Z*_α_ = 1.96) for this study. S is the standard deviation estimated from the population, derived from a previous study (15) showing that the standard deviation of daily energy intake for pregnant women during the third trimester was 408.2 kcal. d is the maximum standard error, which we determined to be 50 kcal. It was calculated that a minimum of 257 pregnant women were needed in the second and third trimesters, respectively, to ensure adequate power.

### 2.2 Dietary data collection and analysis

Data collection was delegated to a third-party organization, Taylor Nelson Sofres (Shanghai, China). With the help of Tablet/iPad/Phone resources, an online diary was utilized to record the dietary intake of pregnant women over 4 days, including two workdays and two weekend days. This online diary contained a food list covering 328 different types of food and beverages. To assist participants in accurately estimating their dietary intake, a food atlas featuring 303 types of food and two types of fluid containers were also provided as visual aids. Detailed explanations have been presented in our previous report (6). Before conducting the study, all investigators must receive training on survey techniques. To verify the feasibility and scientific validity of the online diary, a pre-survey was conducted. To ensure the authenticity and reliability of the dietary data and avoid duplication, investigators provided guidance and assisted participants in using the online diary. The raw data retrieved from the original records were promptly reviewed and scrutinized for the completeness and validity of the results.

The daily consumption of each kind of food was calculated, with the food in its typical state and 100% edible. For example, vegetables and fruits were in their fresh state, while cereal and their products were in their dry state. For compound processed foods (such as dumplings, steamed stuffed buns, pizza, burger, sandwich, etc.), each component was recorded and the proportion of each component was calculated. For example, in a chicken burger, bread, chicken, and vegetables were all calculated separately. Then, food were divided into 8 categories: staple food (cereal and their products, potatoes and beans other than soybeans); vegetables; fruits; animal-based food (livestock meat, poultry, fish, shrimp, shellfish and eggs); milk and its products; soybean and its products; nuts; and cooking oil. The total daily consumption of each category of food was calculated and compared with the recommended intake in the Chinese balanced dietary pagoda for pregnant women during different pregnancy periods (16).

The daily intake of energy and key nutrients was calculated using the China Food Composition Tables (17). Foods with incomplete data were supplemented by the National Nutrient Database of the United States Department of Agriculture (18) or be replaced with similar foods. Nutritional supplements were also investigated (Table 1) and calculated based on the nutrient content indicated in the product manual. In accordance with the 2023 Chinese Dietary Reference Intakes (DRIs) (19), the intake of energy and key nutrients among pregnant women during different trimesters were evaluated. The energy measurements were derived from the estimated energy reference (EER) value. The indicator for carbohydrates was the Estimated Average Requirement (EAR). Furthermore, for both carbohydrates and fats, the Acceptable Macronutrient Distribution Ranges (AMDR) were utilized to establish the lower and upper bounds of their contribution to total energy intake. The lower limits of vitamin E were established using adequate intake (AI) value. Protein, minerals and other vitamins were determined by the EAR and recommended nutrient intake (RNI), while the upper limits for these nutrients were based on the tolerable upper intake level (UL) values.

**Table 1:** The usage of nutritional supplements of Chinese pregnant women in 2018 (*n* = 653).

Types of nutritional supplements	*n* (%)
Multiple micronutrients	111 (17.0)
Folic acid	82 (12.6)
DHA	58 (8.9)
Multiple vitamins	54 (8.3)
Iron	20 (3.1)
Calcium	11 (1.7)
Vitamin D	8 (1.2)
Others	15 (2.3)

### 2.3 Statistical analysis

All data were analyzed using the SPSS software package version 26.0 (IBM, New York, NY, USA). Pregnant women of different pregnancy periods were grouped according to their residence. After conducting the Shapiro–wilk test, the intake of various food categories, energy, and key nutrients exhibited a non-normal distribution, and was therefore represented by median (*P*_25_; *P*_75_), and the Mann–Whitney U test was used to compare the differences. The evaluation results of dietary structure, energy, and key nutrients was presented as number and percentage [*n* (%)], and Chi-square test was used to compare the differences. *p* < 0.05 is used as the criterion for statistical significance of the differences.

## 3 Result

### 3.1 Age and pregnancy distribution of the study participants

A total of 653 pregnant women participated in this study, of which 307 were in the second trimester (mean age: 28.4 ± 3.9 years, age range: 20 ~ 41 years), and 346 were in the third trimester (mean age: 28.6 ± 3.8 years, age range: 20 ~ 44 years) (Supplementary Table S1). The mean age of urban pregnant women during the second and third trimesters was significantly higher than that of rural ones, respectively (*p* < 0.001). Additionally, the difference in age distribution among urban and rural pregnant women in the third trimester was also statistically significant. (*p* < 0.05) (Table 2).

**Table 2:** The age distribution of pregnant women across urban (*n* = 372) and rural (*n* = 281) China in 2018.

Category	Pregnant women (*n* = 653)					
	Second trimester (*n* = 307)			Third trimester (*n* = 346)		
	Urban	Rural	*p* ^1^	Urban	Rural	*p* ^1^
Sample size	179	128		193	153	
Age (years), mean (±SD)	29.1 ± 4.1	27.3 ± 3.4	**0.000**	29.3 ± 3.9	27.7 ± 3.5	**0.000**
(Min, Max), years	20, 40	20, 41		21, 44	20, 41	
<25, *n* (%)	31 (17.3)	32 (25)		27 (14)	38 (24.8)	
25–35, *n* (%)	133 (74.3)	91 (71.1)	0.293	151 (78.2)	108 (70.6)	**0.043**
>35, *n* (%)	15 (8.4)	5 (3.9)		15 (7.8)	7 (4.6)	

^1^A statistically significant difference emerged between urban and rural areas (*p* < 0.05), determined through either a two-independent sample *t*-test or Chi-square test.

Bold values indicate *p* < 0.05.

### 3.2 Intake of foods from different categories

The staple food typically comprises cereal and their products, potatoes and beans other than soybeans. Whether in the second or third trimester, the consumption of staple food among rural pregnant women was higher than that of urban ones, with a statistically significant difference observed during the second trimester (*p* < 0.05) (Table 3). Furthermore, in the second trimester, 39.6% of urban pregnant women had an consumption of staple food higher than the recommended value, whereas this proportion rises to 54.7% in rural areas, with the difference being statistically significant (*p* < 0.005) (Table 4). However, the consumption of potatoes among urban and rural pregnant women was inadequate, with approximately 90.0% of the population consuming less than the recommended value in the second or third trimester (Table 4).

**Table 3:** Dietary structures of pregnant women across urban (*n* = 372) and rural (*n* = 281) China in 2018.

Food categories (g/d)	Second trimester			Third trimester		
	Urban (*n* = 179)	Rural (*n* = 128)	*p* ^1^	Urban (*n* = 193)	Rural (*n* = 153)	*p* ^1^
**Cereal and their products, potatoes and beans other than soybeans**	221.7 (163.7; 288.8)	258.5 (176.3; 327.5)	**0.022**	223.1 (165.7; 292.6)	247.4 (174.2; 310.7)	0.308
Potatoes	18.5 (0.0; 42.4)	17.1 (0.0; 41.9)	0.169	22.4 (0.0; 47.5)	13.3 (0.0; 35.6)	0.060
**Vegetables**	159.1 (97.9; 239.6)	121.5 (70.0; 200.9)	**0.006**	169.5 (104.9; 236.1)	114.9 (67.1; 188.7)	**0.000**
Green leafy vegetables and colored vegetables such as red and yellow	81.7 (40.4;136.6)	67.9 (35.9; 106.6)	0.070	87.5 (52.9; 129.9)	65.9 (38.2; 98.5)	**0.000**
**Fruits**	188.7 (119.7; 310.8)	169.6 (90.8; 269.3)	0.099	173.1 (111.0; 277.0)	160.5 (73.5; 248.4)	**0.034**
**Livestock meat, poultry, fish, shrimp, shellfish and egg**	173.0 (130.3; 246.4)	164.6 (110.4; 223.5)	0.198	184.8 (132.1; 250.6)	166.8 (106.5; 231.3)	0.094
Livestock meat and poultry	91.2 (59.5; 146.4)	85.7 (51.0; 141.8)	0.538	94.8 (56.2; 140.2)	91.4 (61.1; 146.7)	0.890
Fish, shrimp and shellfish	20.2 (3.6; 52.2)	19.3 (0.0; 44.0)	0.275	27.2 (7.1; 58.0)	15.3 (0.0; 43.7)	**0.008**
Egg	45.5 (26.1; 63.6)	40.6 (24.0; 62.9)	0.191	44.6 (28.3; 64.4)	39.4 (22.4; 61.1)	0.120
**Milk and its products**	185.9 (64.0; 293.6)	150.1 (75.5; 211.3)	0.050	176.4 (87.3; 305.3)	135.7 (50.0; 250.0)	**0.014**
**Soybean and its products**	4.4 (0.0; 8.8)	3.6 (0.0; 11.2)	0.855	5.8 (1.4; 11.9)	4.1 (0.0; 13.5)	0.556
**Nuts**	9.3 (0.0; 22.9)	5.1 (0.0; 17.2)	**0.029**	7.5 (0.0; 20.0)	6.5 (0.0; 19.0)	0.761
**Cooking oil**	30.4 (27.0; 33.5)	30.0 (26.5; 32.8)	0.241	30.3 (26.5; 33.5)	30.0 (26.3; 32.1)	0.161

The data are expressed as median (*P*_25_; *P*_75_). ^1^A statistically significant difference emerged between urban and rural areas (*p* < 0.05), determined through Mann–Whitney U test.

Bold values indicate *p* < 0.05.

**Table 4:** The comparative analysis of dietary recommendations and actual food intake from different categories in pregnant women across urban (*n* = 372) and rural (*n* = 281) China in 2018.

Food categories (g/d)	Second trimester						*p* ^4^	Third trimester						*p* ^4^
	Urban (*n* = 179)			Rural (*n* = 128)				Urban (*n* = 193)			Rural (*n* = 153)			
	Below rec^1^	Above rec^2^	Rec^3^	Below rec^1^	Above rec^2^	Rec^3^		Below rec^1^	Above rec^2^	Rec^3^	Below rec^1^	Above rec^2^	Rec^3^	
**Cereal and their products, potatoes and beans other than soybeans**	73 (40.8)	71 (39.6)	35 (19.6)	48 (37.5)	70 (54.7)	10 (7.8)	**0.004**	97 (50.3)	63 (32.6)	33 (17.1)	65 (42.5)	63 (41.2)	25 (16.3)	0.242
Potatoes	163 (91.1)	16 (8.9)	–	121 (94.5)	7 (5.5)	–	0.255	170 (88.1)	23 (11.9)	–	141 (92.2)	12 (7.8)	–	0.212
**Vegetables**	174 (97.2)	3 (1.7)	2 (1.1)	125 (97.7)	–	3 (2.3)	0.242	186 (96.4)	3 (1.6)	4 (2.0)	148 (96.7)	1 (0.7)	4 (2.6)	0.702
Green leafy vegetables and colored vegetables such as red and yellow	168 (93.9)	4 (2.2)	7 (3.9)	124 (96.9)	–	4 (3.1)	0.216	177 (91.7)	7 (3.6)	9 (4.7)	149 (97.4)	–	4 (2.6)	**0.033**
**Fruits**	92 (51.4)	48 (26.8)	39 (21.8)	77 (60.2)	22 (17.2)	29 (22.7)	0.129	112 (58.0)	28 (14.5)	53 (27.5)	100 (65.4)	15 (9.8)	38 (24.8)	0.288
**Livestock meat, poultry, fish, shrimp, shellfish and egg**	67 (37.4)	68 (38)	44 (24.6)	53 (41.4)	45 (35.2)	30 (23.4)	0.777	92 (47.7)	70 (36.3)	31 (16.1)	85 (55.6)	41 (26.8)	27 (17.6)	0.169
Livestock meat and poultry	35 (19.6)	112 (62.6)	32 (17.9)	31 (24.2)	74 (57.8)	23 (18)	0.596	35 (18.1)	121 (62.7)	37 (19.2)	26 (17.0)	92 (60.1)	35 (22.9)	0.699
Fish, shrimp and shellfish	132 (73.7)	25 (14.0)	22 (12.3)	100 (78.1)	10 (7.8)	18 (14.1)	0.241	166 (86.0)	9 (4.7)	18 (9.3)	136 (88.9)	3 (2)	14 (9.2)	0.390
Egg	99 (55.3)	80 (44.7)	–	78 (60.9)	50 (39.1)	–	0.325	112 (58)	81 (42)	–	99 (64.7)	54 (35.3)	–	0.206
**Milk and its products**	136 (76.0)	9 (5.0)	34 (19.0)	110 (85.9)	4 (3.1)	14 (10.9)	0.097	142 (73.6)	9 (4.7)	42 (21.8)	125 (81.7)	4 (2.6)	24 (15.7)	0.189
**Soybean and its products**	165 (92.2)	14 (7.8)	–	116 (90.6)	12 (9.4)	–	0.630	174 (90.2)	19 (9.8)	–	130 (85.0)	23 (15.0)	–	0.142
**Nuts**	92 (51.4)	84 (46.9)	3 (1.8)	80 (62.5)	47 (36.7)	1 (0.8)	0.053	107 (55.4)	85 (44.0)	1 (0.5)	90 (58.8)	61 (39.9)	2 (1.3)	0.528
**Cooking oil**	5 (2.8)	165 (92.2)	9 (5.0)	3 (2.3)	116 (90.6)	9 (7.0)	0.807	4 (2.1)	182 (94.3)	7 (3.6)	10 (6.5)	135 (88.2)	8 (5.2)	**0.036**

The data are expressed as number and percentage [*n* (%)]. Recommended values of food categories can be found in Supplementary Table S3. ^1^Below rec: number and percentage of participants with food intakes below the recommended value; ^2^Above rec: number and percentage of participants with food intakes above the recommended value; ^3^Rec: number and percentage of participants with food intakes within the recommended value; ^4^A statistically significant difference emerged between urban and rural areas (*p* < 0.05), determined through Chi-square test.

Bold values indicate *p* < 0.05.

Whether in the second or third trimester, the consumption of vegetables among urban pregnant women was significantly higher than that in rural areas (*p* < 0.05) (Table 3). Nonetheless, the proportion of their consumption below the recommended value was over 95.0% in both areas (Table 4). Further analysis reveals that, although the consumption of green leafy vegetables and colored vegetables among urban pregnant women was significantly higher than that in rural areas (*p* < 0.001) in the third trimester (Table 3), the proportion of their consumption below the recommended value was also over 90.0% (*p* < 0.05) (Table 4). Additionally, the fruit consumption of urban pregnant women was also higher than that in rural areas, with a statistically significant difference observed in the third trimester (*p* < 0.05) (Table 3). However, the difference in the proportion of their fruit consumption below the recommended value between the two areas was not statistically significant during either the second or third trimester (Table 4).

Although the consumption of livestock meat, poultry, fish, shrimp, shellfish and eggs among urban pregnant women was higher than that in rural areas, only the difference in consumption of fish, shrimp, and shellfish in the third trimester was statistically significant (*p* < 0.05) (Table 3). The comparative analysis showed that the proportion of their consumption of livestock meat and poultry exceeding the recommended values in urban areas was over 62.0%, while in rural areas, it was around 59.0%. Moreover, in both urban and rural areas, the proportion of pregnant women whose consumption of fish, shrimp, and shellfish was below the recommended values exceeded 70.0 and 85.0% in the second and third trimesters, respectively, and the difference between the two areas was not statistically significant (Table 4).

The consumption of milk among urban pregnant women was significantly higher than that in rural areas during the second and third trimesters (*p* ≤ 0.05) (Table 3). However, even in urban areas, the proportion of pregnant women with milk consumption below the recommended value exceeded 70.0%, while in rural areas it exceeded 80.0%, and the difference between the two areas was not statistically significant (Table 4). Similarly, the consumption of soybean was at a low level among pregnant women in both areas, with over 90.0% of urban pregnant women and over 85.0% of rural pregnant women whose consumption of soybean was below the recommended value, and the difference is not statistically significant (Table 4). The consumption of nuts among urban pregnant women was higher than that in rural areas, especially in the second trimester (*p* < 0.05) (Table 3). As for the consumption of cooking oil, there was no statistically significant difference between urban and rural areas (Table 3). However, in the third trimester, the proportion of urban pregnant women whose consumption of cooking oil exceeded the recommended value (94.3%) was significantly higher than that in rural areas (88.2%) (*p* < 0.05) (Table 4).

The food consumption of total population in the second and third trimester of pregnancy was detailed in Supplementary Table S2.

### 3.3 Intake of energy, carbohydrates, protein, and fats

As shown in Table 5, the intake of energy, carbohydrates, protein and fats among urban pregnant women during the second and third trimesters were higher than those in rural areas. Specifically, the differences in energy and protein intake during the second trimester, as well as differences in energy, carbohydrates, and protein intake during the third trimester, were found to be statistically significant (*p* < 0.05). Comparative analysis showed that, in urban areas, the proportion of pregnant women with energy intake below the EER value exceeded 70.0%, while in rural areas it exceeded 80.0%; the difference between the two areas was not statistically significant. During the second trimester, more than 60.0% of rural pregnant women had protein intake below the EAR value, while during the third trimester, more than 70.0% of both urban and rural pregnant women had protein intake below the EAR value in the third trimester, with no significant statistical difference observed (Table 6).

**Table 5:** The intake of energy and nutrients in pregnant women across urban (*n* = 372) and rural (*n* = 281) China in 2018.

Energy and nutrients	Second trimester			Third trimester		
	Urban (*n* = 179)	Rural (*n* = 128)	*p* ^1^	Urban (*n* = 193)	Rural (*n* = 153)	*p* ^1^
Energy (kcal/d)	1676.0 (1374.5; 1992.7)	1525.0 (1233.5; 1872.7)	**0.010**	1626.8 (1385.5; 2037.1)	1522.3 (1263.7; 1792.7)	**0.005**
Carbohydrate (g/d)	184.9 (148.5; 227.0)	177.4 (137.0; 217.0)	0.108	186.7 (149.6; 229.8)	171.0 (130.0; 212.1)	**0.035**
Protein (g/d)	60.4 (47.8; 75.4)	54.0 (41.4; 71.3)	**0.047**	60.3 (48.3; 76.3)	56.2 (44.6; 72.0)	**0.019**
Fat (g/d)	74.6 (60.6; 90.5)	67.6 (55.3; 84.1)	0.453	74.4 (63.0; 86.9)	70.4 (56.4; 81.9)	0.151
SFA (g/d)	16.6 (12.0; 20.5)	15.0 (11.6; 20.1)	0.358	17.2 (12.8; 20.9)	15.6 (12.1; 18.8)	0.170
MUFA (g/d)	31.8 (27.2; 37.3)	29.2 (24.6; 35.1)	0.574	31.5 (27.0; 36.7)	29.8 (25.7; 34.8)	0.106
PUFA (g/d)	14.8 (12.2; 19.5)	13.3 (11.1; 16.4)	0.531	14.6 (12.1; 18.2)	13.4 (11.4; 17.1)	0.605
VA (μgRAE/d)	412.2 (277.3; 679.2)	332.0 (218.0; 535.4)	**0.005**	386.0 (257.7; 628.7)	304.0 (216.8; 445.2)	**0.000**
VB_1_ (mg/d)	0.8 (0.5; 1.5)	0.6 (0.4; 0.8)	**0.000**	0.7 (0.5; 1.4)	0.6 (0.4; 0.8)	**0.000**
VB_2_ (mg/d)	1.1 (0.8; 2.0)	0.9 (0.6; 1.3)	**0.000**	1.0 (0.7; 1.9)	0.9 (0.6; 1.2)	**0.000**
VB_3_ (mgNE/d)	16.2 (11.3; 21.8)	13.8 (10.2; 20.2)	**0.011**	15.5 (10.7; 23.2)	13.6 (10.3; 17.5)	**0.012**
VB_6_ (mg/d)	1.8 (1.2; 2.7)	1.4 (1.0; 2.1)	**0.000**	1.6 (1.2; 2.5)	1.4 (1.0; 1.9)	**0.000**
VB_9_ (μgDFE/d)	231.7 (174.4; 303.7)	196.4 (144.4; 269.7)	**0.011**	239.4 (189.3; 318.7)	197.0 (138.6; 272.8)	**0.000**
VB_12_ (μg/d)	3.1 (2.0; 5.0)	2.8 (1.8; 4.2)	0.170	3.1 (1.8; 5.1)	2.9 (1.7; 4.8)	0.351
VC (mg/d)	117.7 (68.3; 176.2)	66.8 (40.6; 122.5)	**0.000**	101.9 (61.6; 166.5)	73.2 (35.5–134.3)	**0.000**
VE (mg α-TE/d)	34.0 (27.0; 46.0)	29.1 (24.4; 36.0)	**0.000**	32.5 (27.0; 42.8)	28.4 (24.3; 35.2)	**0.000**
Calcium (mg/d)	541.7 (382.0; 822.1)	448.8 (307.2; 625.0)	**0.000**	573.5 (401.2; 857.3)	443.8 (292.1; 606.6)	**0.000**
Iron (mg/d)	21.6 (15.4; 33.1)	16.9 (13.1; 25.7)	**0.000**	20.1 (14.7; 29.3)	17.0 (13.9; 22.0)	**0.001**
Zinc (mg/d)	10.9 (7.4; 14.7)	9.0 (6.7; 12.2)	**0.002**	10.3 (7.3; 14.3)	8.8 (6.6; 11.7)	**0.002**
Magnesium (mg/d)	279.0 (192.7; 360.4)	226.5 (166.2; 300.8)	**0.001**	268.2 (190.6; 365.3)	236.7 (160.9; 319.5)	**0.003**
Phosphorus (mg/d)	867.3 (666.4; 1107.5)	781.1 (601.9; 989.0)	**0.003**	853.1 (677.8; 1090.7)	786.0 (615.4; 990.0)	**0.013**
Iodine (μg/d)	48.6 (22.8; 332.5)	30.5 (19.3; 297.1)	0.174	65.8 (30.0; 445.5)	37.8 (18.9; 237.3)	**0.001**

The data are expressed as median (*P*_25_; *P*_75_). ^1^A statistically significant difference emerged between urban and rural areas (*p* < 0.05), determined through Mann–Whitney U test. SFA, saturated fatty acid; MUFA, monounsaturated fatty acid; PUFA, polyunsaturated fatty acid; VA, vitamin A; RAE, retinol activity equivalent; VB_1_, vitamin B_1_; VB_2_, vitamin B_2_; VB_3_, vitamin B_3_; NE, nicotinic acid equivalent; VB_6_, vitamin B_6_; VB_9_, vitamin B_9_; DFE, dietary folate equivalent; VB_12_, vitamin B_12_; VC, vitamin C; VE, vitamin E; TE, tocopherol equivalent.

Bold values indicate *p* < 0.05.

**Table 6:** The comparative analysis of the recommended values and actual intake of energy and nutrients in pregnant women across urban (*n* = 372) and rural (*n* = 281) China in 2018.

Energy and nutrients	Second trimester				*p* ^5^	Third trimester				*p* ^5^
	Urban (*n* = 179)		Rural (*n* = 128)			Urban (*n* = 193)		Rural (*n* = 153)		
	Below EAR^1^/EER^2^	Above RNI^3^/AI^4^	Below EAR^1^/EER^2^	Above RNI^3^/AI^4^		Below EAR^1^/EER^2^	Above RNI^3^/AI^4^	Below EAR^1^/EER^2^	Above RNI^3^/AI^4^	
Energy	131 (73.2)	–	104 (81.3)	–	0.100	156 (80.8)	–	132 (86.3)	–	0.178
Carbohydrate	38 (21.2)	–	35 (27.3)	–	0.215	59 (30.6)	–	57 (37.3)	–	0.191
Protein	88 (49.2)	56 (31.3)	77 (60.2)	36 (28.1)	0.093	144 (74.6)	34 (17.6)	119 (77.8)	18 (11.8)	0.254
VA	112 (62.6)	37 (20.7)	94 (73.4)	18 (14.1)	0.133	130 (67.4)	38 (19.7)	126 (82.4)	17 (11.1)	**0.007**
VB_1_	118 (65.9)	46 (25.7)	107 (83.6)	14 (10.9)	**0.002**	140 (72.5)	43 (46.2)	136 (88.9)	15 (15.7)	**0.001**
VB_2_	87 (48.6)	78 (43.6)	87 (68.0)	30 (23.4)	**0.001**	113 (58.5)	68 (35.2)	122 (79.7)	25 (9.8)	**0.000**
VB_3_	30 (16.8)	129 (72.1)	30 (23.4)	77 (60.2)	0.090	40 (20.7)	127 (65.8)	35 (22.9)	93 (62.7)	0.608
VB_6_	100 (55.9)	65 (36.3)	90 (70.3)	30 (23.4)	**0.034**	115 (59.6)	65 (33.7)	116 (75.8)	27 (60.8)	**0.003**
VB_9_	171 (95.6)	3 (1.8)	127 (99.2)	1 (0.8)	0.126	188 (97.4)	4 (2.1)	150 (98.0)	1 (0.7)	0.405
VB_12_	63(35.2)	93 (52.0)	51 (39.8)	62 (48.4)	0.707	76 (39.4)	103 (53.4)	66 (43.1)	78 (51.0)	0.730
VC	69 (38.5)	93 (52.0)	76 (59.4)	36 (28.1)	**0.000**	91 (47.2)	81 (42.0)	93 (60.8)	47 (30.7)	**0.041**
VE	–	179 (100)	–	128 (100)	–	–	193 (100)	–	153 (100)	–
Calcium	108 (60.3)	51 (28.5)	100 (78.1)	13 (10.2)	**0.000**	114 (59.1)	51 (26.4)	120 (78.4)	17 (11.1)	**0.000**
Iron	74 (41.3)	69 (38.5)	74 (57.8)	34 (26.6)	**0.016**	111 (57.5)	50 (25.9)	115 (75.2)	25 (16.3)	**0.003**
Zinc	59(33.0)	92 (51.4)	58 (45.3)	48 (37.5)	**0.044**	73 (37.8)	91 (47.2)	72 (47.1)	54 (35.3)	0.084
Magnesium	102 (57.0)	41 (22.9)	96 (75.0)	16 (12.5)	**0.008**	114 (59.1)	46 (23.8)	108 (70.6)	22 (14.4)	0.088
Phosphorus	34 (19.0)	118 (65.9)	31 (24.2)	74 (57.8)	0.345	26 (13.5)	131 (67.9)	35 (22.9)	96 (62.7)	0.062
Iodine	125 (69.8)	50 (27.9)	91 (71.1)	34 (26.6)	0.965	113 (58.5)	64 (33.2)	109 (71.2)	39 (25.5)	0.299

The data are expressed as number and percentage [*n* (%)]. Recommended values of energy and nutrients can be found in Supplementary Table S6. ^1^Below EAR: number and percentage of participants with intakes of carbohydrate, protein, vitamins A, B_1_, B_2_, B_3_, B_6_, B_9_, B_12_, C, and minerals calcium, iron, zinc, magnesium, phosphorus, and iodine below the EAR. ^2^Below EER: number and percentage of participants with energy intake below the EER. ^3^Above RNI: number and percentage of participants with intake levels of VA, VB_1_, VB_2_, VB_3_, VB_6_, VB_9_, VB_12_, VC, calcium, iron, zinc, magnesium, phosphorus, and iodine above the RNI values. ^4^Above AI: number and percentage of participants with intake of VE above the AI value. ^5^A statistically significant difference emerged between urban and rural areas (*p* < 0.05), determined through Chi-square test. EAR, estimated average requirement; EER, estimated energy reference; RNI, recommended nutrient intake; AI, adequate intake; VA, vitamin A; VB_1_, Vitamin B_1_; VB_2_, vitamin B_2_; VB_3_, vitamin B_3_; VB_6_, vitamin B_6_; VB_9_, vitamin B_9_; VB_12_, vitamin B_12_; VC, vitamin C; VE, vitamin E.

Bold values indicate *p* < 0.05.

Table 7 shows the contribution of carbohydrate, fat, and protein to total energy intake, with no statistically significant differences between urban and rural areas during different trimesters of pregnancy. Regardless of whether it was the second or third trimester, the proportion of both urban and rural participants whose energy intake from carbohydrates was below the AMDR value was over 70.0%, while the proportion of participants whose energy intake from fats was above the AMDR value was over 80.0%. There was also no statistically significant difference between urban and rural areas during different trimesters of pregnancy.

**Table 7:** Energy ratios from carbohydrates, fat and proteins in pregnant women across urban (*n* = 372) and rural (*n* = 281) China in 2018.

Energy ratio (%)	Second trimester (*n* = 307)					Third trimester (*n* = 346)				
		Median (*P*_25_; *P*_75_)	Below AMDR^a^	Within AMDR^b^	Above AMDR^c^		Median (*P*_25_; *P*_75_)	Below AMDR^a^	Within AMDR^b^	Above AMDR^c^
Carbohydrate	Urban (*n* = 179)	45.0 (40.8; 48.6)	143 (79.9)	33 (18.4)	3 (1.7)	Urban (*n* = 193)	44.5 (39.9; 50.2)	144 (74.6)	42 (21.8)	7 (3.6)
	Rural (*n* = 128)	46.1 (40.2; 51.1)	92 (71.9)	31 (24.2)	5 (3.9)	Rural (*n* = 153)	45.6 (40.3; 50.0)	115 (74.6)	31 (21.8)	7 (3.6)
	*p* ^1^	0.332				*p* ^1^	0.681			
Fat	Urban (*n* = 179)	40.4 (37.2; 45.5)	7 (3.9)	13 (11.7)	128 (84.4)	Urban (*n* = 193)	41.0 (36.2; 45.6)	14 (7.3)	24 (12.4)	155 (80.3)
	Rural (*n* = 128)	40.8 (36.4; 45.1)	7 (5.5)	13 (10.2)	108 (84.4)	Rural (*n* = 153)	41.1 (36.3; 45.5)	4 (2.6)	25 (16.3)	124 (81.0)
	*p* ^1^	0.857				*p* ^1^	0.630			
Protein	Urban (*n* = 179)	14.4 (13.0; 16.0)	–	–	–	Urban (*n* = 193)	14.8 (13.2; 16.7)	–	–	–
	Rural (*n* = 128)	14.1 (12.6; 16.7)	–	–	–	Rural (*n* = 153)	14.4 (13.0; 16.7)	–	–	–
	*p* ^1^	0.728				*p* ^1^	0.521			

The data are expressed number and percentage [*n* (%)]. AMDR: acceptable macronutrient distribution range. The AMDR values for pregnant women are 50–65%E for carbohydrate and 20–30%E for fat. %E denotes the nutrient’s contribution to total energy expressed as a percentage. ^a^Below AMDR: number and proportion of participants with macronutrient-derived energy ratios below the AMDR. ^b^Within AMDR: number and proportion of participants with macronutrient-derived energy ratios within the AMDR. ^c^Above AMDR: number and proportion of participants with macronutrient-derived energy ratios above the AMDR. ^1^A statistically significant difference emerged between urban and rural areas (*p* < 0.05), determined through Mann–Whitney U test.

The intake of energy and macronutrients, along with the energy ratios from carbohydrates, fat and proteins by the total population in the second and third trimesters of pregnancy was detailed in Supplementary Tables S4, S5.

### 3.4 Intake of vitamins and minerals

Except for vitamin B_12_, the intakes of vitamins among urban pregnant women during the second and third trimesters were higher than those in rural areas, and these differences were statistically significant (*p* < 0.05) (Table 5). After comparative analysis, the results showed that, in urban areas, the proportion of pregnant women during the second trimester whose intake of vitamin A, vitamin B_1_, vitamin B_6_, and vitamin B_9_ was below the EAR values surpassed 50.0%, and this inadequacy extended to include vitamin B_2_ in the third trimester as well (Table 6). In rural areas, the proportion of pregnant women whose intake of vitamin A, vitamin B_1_, vitamin B_2_, vitamin B_6_, vitamin B_9_, and vitamin C was below the EAR values also exceeded 50.0% during both the second and third trimesters (Table 6). It is particularly noteworthy that, whether in the second or third trimester, the proportion of both urban and rural pregnant women whose intake of vitamin B_9_ was below the EAR values exceeded 95.0% (Table 6). When comparing different areas, during the second trimester, the proportion of rural pregnant women whose intake of vitamin B_1_, vitamin B_2_, vitamin B_6_, and vitamin C was below the EAR values was higher than that in urban areas, and these differences were statistically significant (*p* < 0.05) (Table 6). In the third trimester, the proportion of rural pregnant women whose intake of vitamin A, vitamin B_1_, vitamin B_2_, vitamin B_6_, and vitamin C was below the EAR values was also higher than that in urban areas, and these difference were also statistically significant (*p* < 0.05) (Table 6).

Except for iodine intake during the second trimester, the intakes of minerals among urban pregnant women during the second and third trimesters were higher than those observed in rural areas, with statistically significant differences (*p* < 0.05) (Table 5). Comparative analysis showed that in urban areas, the proportion of pregnant women in the second trimester whose intake of calcium, magnesium, and iodine was below the EAR values exceeded 50.0%, and this inadequacy extended to include iron in the third trimester as well (Table 6). In rural areas, the proportion of pregnant women whose intake of calcium, iron, magnesium, and iodine during the second and third trimesters was below the EAR values also exceeded 50.0% (Table 6). When comparing different areas, during the second trimester, the proportion of rural pregnant women whose intake of calcium, iron, zinc, and magnesium was below the EAR values was higher than that in urban areas, and these differences were statistically significant (*p* < 0.05) (Table 6). In the third trimester, the proportion of rural pregnant women whose intake of calcium and iron was below the EAR values was also higher than that in urban areas, and these difference were also statistically significant (*p* < 0.01) (Table 6).

The intake of vitamins and minerals by the total population in the second and third trimesters of pregnancy was detailed in Supplementary Table S4.

## 4 Discussion

Herein, utilizing a specialized dietary survey method, we conducted a nationwide survey in China to comprehensively compare the dietary structures and the intake of energy and nutrients from both diets and nutritional supplements among pregnant women during their second and third trimesters, with a focus on the differences between urban and rural areas. Our study surpasses previous studies in two major aspects: a nationally representative and equitably distributed sample of pregnant women, ensuring representation of the Chinese pregnant population; and a specialized dietary survey method that significantly improves the accuracy of dietary intake estimation. Our findings can provide a scientific foundation for dietary guidance and interventions for Chinese pregnant women.

In this study, we found that pregnant women during the second and third trimesters suffered from inadequate energy intake, the proportion of urban and rural pregnant women with energy intake below the EER value exceeded 70.0%. In contrast, a study involving 793 pregnant women in Spain showed that 82.6% of pregnant women had adequate energy intake in the third trimester (15). This phenomenon may be attributed to the difference in evaluation standards between different countries, as well as underestimation of cooking oil consumption and neglect of additional meals due to the complexity of Chinese cuisine. These are common challenges encountered in Chinese dietary surveys. Moreover, this group also had inadequate protein intake, especially noticeable during the third trimester of pregnancy. Analysis of food categories revealed that the inadequate consumption of fish, shrimp and shellfish, milk and its products, and soybean and its products were key contributors. In terms of energy ratios, this study revealed that over 70.0% of pregnant women derived less than 50.0% of their energy from carbohydrates, while more than 80.0% obtained over 30.0% of energy from fats. These findings aligned with a study of 1,652 individuals in Chengdu City, where the proportion of pregnant women with energy intake from fat exceeding 30.0% in the second and third trimesters was 77.6 and 82.9%, respectively (20). By analyzing the consumption of foods from different categories in this study, the reason for the above phenomenon is that this group has inadequate consumption of potatoes and vegetables, and excessive consumption of livestock meat and poultry and cooking oil. This dietary imbalance is not only a matter of personal choice, but also closely related to socioeconomic determinants. For instance, the intake of high-quality protein sources (such as fish and dairy products) and diverse vegetables in rural areas was significantly lower than that in urban areas, which may be related to food affordability, availability in local markets, and the dietary knowledge popularization. Based on these findings, it is recommended that urban and rural pregnant women in the second and third trimesters of pregnancy in China increase their consumption of potatoes, vegetables, fish, shrimp and shellfish, milk and its products, as well as soybeans and its products, while reducing their consumption of livestock meat and poultry and cooking oil to ensure a balanced dietary structure during pregnancy.

In this study, after investigating diets and nutritional supplements, the inadequate intake of vitamin A and B vitamins was observed among both urban and rural pregnant women. Notably, the nutritional status of rural pregnant women in the third trimester was particularly concerning. According to the 2015 CHNS data, although there was a significant improvement in the vitamin A nutritional status among rural pregnant women in China, attention should still be paid to the third trimester, with a deficiency rate of 2.1% and a marginal deficiency rate of 14.2% (21). Our result was consistent with this, finding that the proportion of rural pregnant women in the third trimester with inadequate vitamin A intake was 82.4%. The main sources of vitamin A are animal liver and dark-green leafy vegetables and fruits (19). If these foods were not consumed during the survey period, there would be a substantial variation in vitamin A intake among pregnant women. Therefore, it is recommended that pregnant women’s diets include animal liver 1 ~ 2 times per week to increase vitamin A intake (16). In our study, the intakes of B vitamins (excluding vitamin B_3_ and vitamin B_12_) were generally not ideal among pregnant women, especially in rural areas. In daily diet, vitamin B_1_ mainly comes from cereal and its products, but as the refinement level of cereal processing increases, the content of vitamin B_1_ gradually decreases (19). In our study, the consumption of cereal and its products among pregnant women remained normal, so the inadequate intake of vitamin B_1_ may be related to the overly refined processing of cereal and its products in this population. As for vitamin B_9_, its main sources are animal liver and green vegetables (19). More than 95.0% of pregnant women in this survey have inadequate intake of vitamin B_9_, which may be partly due to their inadequate intake of these foods. Due to the easy degradation of vitamin B_9_ during food storage or cooking, it is difficult to meet the needs of pregnant women solely relying on food sources (16). Therefore, taking additional supplements is particularly crucial on top on a balanced diet. In China, more attention has been paid to taking vitamin B_9_ supplements before and during the first trimester of pregnancy to prevent neural tube defects. A cross-sectional study in Shanghai showed that 93.4% (14585/15615) of pregnant women in the first trimester took vitamin B_9_ supplements (22). However, recent studies, including in China, advocate for the continued supplementation of vitamin B_9_ or multivitamins containing vitamin B_9_ by pregnant women during the second and third trimesters for the well-being of pregnant women and their fetuses (23, 24). Lack of vitamin B_9_ in the second and third trimesters of pregnancy may lead to megaloblastic anemia, increase the risk of gestational hypertension, and be associated with placental abruption, fetal growth restriction, premature birth, etc. (25). These are still common pregnancy complications and adverse pregnancy outcomes among Chinese pregnant women. Therefore, further guidance is needed for pregnant women to supplement vitamin B_9_ in the second and third trimesters, whether in rural or urban areas of China. In addition, the urban–rural differences in intake of vitamin A and B vitamins highlight the impact of education level and medical accessibility. Public health measures need to be precisely designed to address these socioeconomic barriers.

In terms of mineral intake, our study found that pregnant women in the second and third trimesters experienced inadequate intake of calcium, magnesium, and iodine, with this issue being particularly severe in rural areas. Inadequate calcium intake during pregnancy may lead to maternal complications, such as muscle cramps and gestational hypertension, and may have adverse effects on fetal growth, development and bone formation (16, 26, 27), which is a key concern for Chinese pregnant women and newborns. Our study found that the daily calcium intake of pregnant women during the second and third trimesters decreased, with more than 78.0% of rural pregnant women having calcium intakes below the EAR. In contrast, a survey conducted in the United States among 1,100 pregnant women found that less than 20.0% of participants had calcium intake from diet and nutritional supplements below the EAR, primarily due to the widespread habit of consuming milk and its products among American pregnant women (28). Given that milk and its products are the best source of dietary calcium, promoting its consumption among Chinese pregnant women remains a critical step in addressing this issue (16). Previous dietary surveys of pregnant women in Spain, Japan, and certain regions of China have rarely focused on magnesium intake (10, 11, 15, 29). In our study, however, it also emerged as a concern, with more than 70.0% of rural pregnant women having its intake below the EAR during the second and third trimesters. Similar findings were reported in the United States, where 34.1 ~ 41.7% of pregnant women had dietary magnesium intake below the EAR, a figure that only modestly improved to 24.4 ~ 34.6% with nutritional supplementation (28). This suggests that supplements cannot bridge the magnesium gap in dietary intake. Magnesium, a cofactor for enzymes involved in energy metabolism, protein synthesis, and DNA and RNA synthesis, is an essential mineral for maintaining maternal health and fetal growth and development (30). To ensure its adequate intake, pregnant women are encouraged to consume magnesium-rich foods such as whole grains, legumes, and nuts (19). Iodine, another essential mineral, plays a critical role in thyroid hormone synthesis (19). Its deficiency in pregnant women not only compromises maternal health but can also impair the intellectual and physical development of the fetus (19). A study of 4,635 pregnant women in Shanghai showed that 54.5% of pregnant women had iodine deficiency (31). While China’s iodized salt policy has been fundamental in reducing iodine deficiency disorders, our study found that 58.5 ~ 71.2% of pregnant women in their second and third trimesters still had inadequate iodine intake, with rural areas facing greater challenges. Given the elevated iodine requirements during pregnancy, relying solely on iodized salt is inadequate. Supplementing through iodine-rich foods, particularly seafood, is essential to meet the increased nutritional demands of this critical physiological stage. Moreover, the persistent inadequacy of key minerals like calcium and iodine, particularly in rural areas, points to systemic issues beyond individual diet choices. These include the economic accessibility of nutrient-dense foods like dairy and seafood, and potentially the variable implementation of food fortification programs. To address these issues, it is necessary to promote nutrition intervention measures at the level of public health policies, strengthen nutrition education in rural areas, and improve the implementation mechanism of food reinforcement projects.

Based on this nationwide survey, we propose multi-level strategies to improve maternal nutrition in China. At the individual level, culturally appropriate health education should be provided, focusing on balanced diets and proper nutrient supplementation, especially for rural pregnant women. At the health service level, antenatal care should include structured dietary assessment, personalized counseling, and locally adapted nutrition education. Healthcare provider training is essential. At the policy and systems level, structural actions should address socioeconomic drivers of dietary inequity. These include enhancing food fortification and targeted supplement distribution in underserved areas, implementing economic measures such as subsidies to improve access to nutritious foods for low-income families, and updating national dietary guidelines and strengthening long-term nutritional monitoring systems.

However, this study has some limitations. Firstly, our study is limited to pregnant women in their second and third trimesters. Women typically confirm their pregnancy 6 ~ 7 weeks after a missed menstrual period. Subsequently, they often experience early pregnancy symptoms such as nausea, vomiting, and reduced appetite. These factors not only make recruiting participants in the first trimester highly challenging but also compromise dietary intake accuracy during this period. Secondly, although a quantitative food atlas was used to improve the accuracy of food weight estimation, the inherent complexity of dietary surveys, communal dining habits, and the intricate diversity of Chinese cuisine, may introduce unavoidable biases into the data reported. Thirdly, the analysis focused on group differences without conducting multivariate adjustment for confounding factors such as income, education level, or parity, which may explain part of the urban–rural gap. Finally, the cross-sectional design precluded causal inference and failed to capture seasonal or trimester-specific dietary variations, thereby limiting longitudinal insight into how maternal diet and nutrient intake affects maternal and infant health outcomes.

## 5 Conclusion

In summary, this nationwide survey provides a comprehensive comparison of dietary structures and nutrient intake among pregnant women in their second and third trimesters in China, highlighting significant gaps in energy, protein, vitamins and minerals between urban and rural areas. Future research should adopt longitudinal designs to establish causality and explore how socioeconomic factors mediate the impact of nutrition on health outcomes. By integrating these individual, service, and policy-level strategies, a synergistic approach can be forged to effectively bridge the urban–rural nutritional gap and improve maternal and infant health nationwide.

## Data Availability

The original contributions presented in the study are included in the article/Supplementary material, further inquiries can be directed to the corresponding authors.
