# Supplementary material for: Differences in nutritional intake from diet and nutritional supplements between urban and rural pregnant women in China: a nationwide cross-sectional study
Source: Front Nutr. 2026 Jan 14;12:1634739. doi: 10.3389/fnut.2025.1634739 (PMC12847047; doi:10.3389/fnut.2025.1634739)
Supplement: Supplementary file 1 [file Table_1.DOCX]

Table S1 Age of Chinese pregnant women in 2018

| **Age** | **Second trimester (n=307)** | **Third trimester (n=346)** |
| --- | --- | --- |
| Mean±SD, years | 28.37±3.915 | 28.63±3.812 |
| (Min, Max), years | 20, 41 | 20, 44 |
| <25, n(%) | 63 (20.5) | 65 (18.8) |
| 25-35, n(%) | 224 (73) | 259 (74.9) |
| >35, n(%) | 20 (6.5) | 22 (6.4) |

Table S2 Food intakes of Chinese pregnant women in the second and third trimester of pregnancy in 2018

| **Food categories (g/d)** | **Second trimester (n=307)** | **Third trimester (n=346)** |
| --- | --- | --- |
| **Cereal and their products, potatoes and beans other than soybeans** | 235.76 (167.93; 312.05) | 234.44 (168.97; 299.94) |
| Potatoes | 18.25 (0; 42.44) | 17.27 (0; 40.67) |
| **Vegetables** | 148.94 (83.11; 221.73) | 149.93 (83.3; 216.01) |
| Green leafy vegetables and colored vegetables such as red and yellow | 75.13 (38.85; 126.45) | 79.49 (45.23; 113.5) |
| **Fruits** | 182.51 (104.26; 286.62) | 167.82 (93.3; 266.65) |
| **Livestock meat, poultry, fish, shrimp, shellfish and egg** | 168.82 (115.65; 237.92) | 172.02 (120.24; 248.51) |
| Livestock meat and poultry | 89.46 (54.95; 143.53) | 93.77 (59.76; 143.53) |
| Fish, shrimp and shellfish | 20.1 (2.01; 48.13) | 22.88 (3.79; 54.4) |
| Egg | 43.81 (25.36; 63.08) | 42.85 (26.1; 63.24) |
| **Milk and its products** | 162.48 (73.7; 268) | 162.48 (75.11; 272.51) |
| **Soybean and its products** | 4.37 (0; 9.13) | 5.22 (1.29; 12.98) |
| **Nuts** | 7.25 (0; 20) | 7.25 (0; 19.8) |
| **Cooking oil** | 30.35 (26.75; 33.31) | 30 (26.45; 32.77) |

The data have been presented as median (*P*_25_; *P*_75_).

Table S3 Recommendations on daily food intake of Chinese pregnant women in the second and third trimester

| **Food categories (g/d)** | **Recommendation** | |
| --- | --- | --- |
|  | **Second trimester** | **Third trimester** |
| **Cereal and their products, potatoes and beans other than soybeans** | 200~250 | 225~275 |
| Potatoes | 75 | 75 |
| **Vegetables** | 400~500 | 400~500 |
| Green leafy vegetables and colored vegetables such as red and yellow | 200~250 | 200~250 |
| **Fruits** | 200~300 | 200~350 |
| **Livestock meat, poultry, fish, shrimp, shellfish and egg** | 150~200 | 175~225 |
| Livestock meat and poultry | 50~75 | 50~75 |
| Fish, shrimp and shellfish | 50~75 | 75~100 |
| Egg | 50 | 50 |
| **Milk and its products** | 300~500 | 300~500 |
| **Soybean and its products** | 20 | 20 |
| **Nuts** | 10 | 10 |
| **Cooking oil** | 25 | 25 |

Table S4 Energy and nutrient intakes of Chinese pregnant women in the second and third trimester of pregnancy in 2018

| **Energy and nutrients** | **Second trimester (n=307)** | **Third trimester (n=346)** |  |
| --- | --- | --- | --- |
| Energy (kcal/d) | 1596.95 (1294.08; 1918.45) | 1582.97 (1323.88; 1917.58) |  |
| Carbohydrate (g/d) | 181.29 (143.32; 222.12) | 181.71 (143.79; 224.5) |  |
| Protein (g/d) | 57.84 (45.15; 74.33) | 58.37 (46.82; 73.97) |  |
| Fat (g/d) | 70.97 (57.77; 88.26) | 72.07 (58.85; 85.46) |  |
| SFA (g/d) | 15.96 (12.38; 20.35) | 16.34 (12.60; 19.78) |  |
| MUFA (g/d) | 30.60 (25.98; 36.80) | 30.80 (25.97; 36.07) |  |
| PUFA (g/d) | 13.82 (11.68; 17.98) | 14.24 (11.82; 17.80) |  |
| Vitamin A (μgRAE/d) | 371.31 (250.72; 596.06) | 344.76 (245.3; 529.82) |  |
| Vitamin B_1_ (mg/d) | 0.66 (0.46; 1.19) | 0.64 (0.45; 1) |  |
| Vitamin B_2_ (mg/d) | 0.99 (0.72; 1.61) | 0.93 (0.67; 1.49) |  |
| Vitamin B_3_ (mgNE/d) | 15.27 (10.72; 21.12) | 14.47 (10.5; 20.67) |  |
| Vitamin B_6_ (mg/d) | 1.59 (1.12; 2.45) | 1.49 (1.11; 2.28) |  |
| Vitamin B_9_ (μgDEF/d) | 220.9 (166.52; 295.48) | 224.17 (167.36; 300.49) |  |
| Vitamin B_12_ (µg/d) | 2.91 (1.9; 4.76) | 3.03 (1.79; 4.93) |  |
| Vitamin C (mg/d) | 100.05 (50.75;160.37) | 90.76 (49.42; 145.19) |  |
| Vitamin E (mg α-TE) | 31 (25.87; 41.81) | 31 (25.7; 39.65) |  |
| Calcium (mg/d) | 496.67 (358.11; 752.34) | 511.55 (352.34; 726.05) |  |
| Iron (mg/d) | 19.56 (14.08; 29.07) | 18.4 (14.3; 26.55) |  |
| Zinc (mg/d) | 10.06 (7.13; 13.83) | 9.47 (7.04; 13.06) |  |
| Magnesium (mg/d) | 259.29 (179.73; 334.11) | 255.07 (177.22; 345.72) |  |
| Phosphorus (mg/d) | 822.24 (627.2; 1064) | 824.31 (653.37; 1052.29) |  |
| Iodine (µg/d) | 41.25 (21.93; 313.6) | 52.72 (23.05; 310.29) |  |

The data have been presented as median (*P*_25_; *P*_75_).

SFA: saturated fatty acid; MUFA: monounsaturated fatty acid; PUFA: polyunsaturated fatty acid; RAE: retinol activity equivalent; NE, nicotinic acid equivalent; DFE: dietary folate equivalent; TE: tocopherol equivalent.

Table S5 Energy ratios from carbohydrates, fat and proteins of Chinese pregnant women in the second and third trimester of pregnancy in 2018

| Energy ratio (%) | **Second trimester (n=307)** | | | |  | **Third trimester (n=346)** | | | |
| --- | --- | --- | --- | --- | --- | --- | --- | --- | --- |
|  | Median  (*P*_25_; *P*_75_) | Below AMDR^a^ | Within AMDR^b^ | Above AMDR^c^ |  | Median  (*P*_25_; *P*_75_) | Below AMDR^a^ | Within AMDR^b^ | Above AMDR^c^ |
| Carbohydrate | 45.27  (40.84; 49.69) | 235  (76.5) | 64  (20.8) | 8  (2.6) |  | 44.88  (40.20; 50.10) | 259  (74.9) | 73  (21.1) | 14  (4.0) |
| Fat | 40.67  (36.74; 45.17) | 14  (4.6) | 34  (11.1) | 259  (84.4) |  | 41.05  (36.28; 45.54) | 18  (5.2) | 49  (14.2) | 179  (80.6) |
| Protein | 14.30  (12.69; 16.23) | - | - | - |  | 14.65  (13.04; 16.71) | - | - | - |

The data have been presented as number and percentage (n (%)).

AMDR: acceptable macronutrient distribution range.

AMDR values for carbohydrate and fat in pregnant women were 50–65%E and 20–30%E, respectively.

%E is the percentage of energy provided by the nutrient as a percentage of total energy.

^a^ Below AMDR: number and percentage of study participants whose intake of energy ratio provided by macronutrients is below the AMDR.

^b^ Within AMDR: number and percentage of study participants whose intake of energy ratio provided by macronutrients is within the AMDR.

^c^ Above AMDR: number and percentage of study participants whose intake of energy ratio provided by macronutrients is above the AMDR.

Table S6 Recommendations on daily energy and nutrient intake of Chinese pregnant women in the second and third trimester

| **Energy and nutrients** | **Second trimester** | |  | **Third trimester** | |
| --- | --- | --- | --- | --- | --- |
|  | **EER or EAR** | **RNI or AI** |  | **EER or EAR** | **RNI or AI** |
| Energy (kcal/d) | 1950 (EER) | - |  | 2100 (EER) | - |
| Carbohydrate (g/d) | 140 | - |  | 155 | - |
| Protein (g/d) | 60 | 70 |  | 75 | 85 |
| Vitamin A (μgRAE/d) | 520 | 730 |  | 520 | 730 |
| Vitamin B_1_ (mg/d) | 1.1 | 1.4 |  | 1.2 | 1.5 |
| Vitamin B_2_ (mg/d) | 1.1 | 1.3 |  | 1.2 | 1.4 |
| Vitamin B_3_ (mgNE/d) | 10 | 12 |  | 10 | 12 |
| Vitamin B_6_ (mg/d) | 1.9 | 2.2 |  | 1.9 | 2.2 |
| Vitamin B_9_ (μgDEF/d) | 520 | 600 |  | 520 | 600 |
| Vitamin B_12_ (µg/d) | 2.4 | 2.9 |  | 2.4 | 2.9 |
| Vitamin C (mg/d) | 95 | 115 |  | 95 | 115 |
| Vitamin E (mg α-TE) | - | 14 (AI) |  | - | 14 (AI) |
| Calcium (mg/d) | 650 | 800 |  | 650 | 800 |
| Iron (mg/d) | 19 | 25 |  | 22 | 29 |
| Zinc (mg/d) | 8.6 | 10.5 |  | 8.6 | 10.5 |
| Magnesium (mg/d) | 300 | 370 |  | 300 | 370 |
| Phosphorus (mg/d) | 600 | 720 |  | 600 | 720 |
| Iodine (μg/d) | 160 | 230 |  | 160 | 230 |

EAR, estimated average reference; EER, estimated energy reference; RNI, recommended nutrient intake; AI, adequate intake; RAE: retinol activity equivalent; NE, nicotinic acid equivalent; DFE: dietary folate equivalent; TE: tocopherol equivalent.
